# The role of the AFD neuron in *C. elegans *thermotaxis analyzed using femtosecond laser ablation

**DOI:** 10.1186/1471-2202-7-30

**Published:** 2006-04-06

**Authors:** Samuel H Chung, Damon A Clark, Christopher V Gabel, Eric Mazur, Aravinthan DT Samuel

**Affiliations:** 1 Division of Engineering and Applied Sciences, Harvard University, Cambridge, MA, USA; 2 Department of Physics, Harvard University, Cambridge, MA, USA

## Abstract

**Background:**

*Caenorhabditis elegans *actively crawls down thermal gradients until it reaches the temperature of its prior cultivation, exhibiting what is called cryophilic movement. Implicit in the worm's performance of cryophilic movement is the ability to detect thermal gradients, and implicit in regulating the performance of cryophilic movement is the ability to compare the current temperature of its surroundings with a stored memory of its cultivation temperature. Several lines of evidence link the AFD sensory neuron to thermotactic behavior, but its precise role is unclear. A current model contends that AFD is part of a thermophilic mechanism for biasing the worm's movement up gradients that counterbalances the cryophilic mechanism for biasing its movement down gradients.

**Results:**

We used tightly-focused femtosecond laser pulses to dissect the AFD neuronal cell bodies and the AFD sensory dendrites in *C. elegans *to investigate their contribution to cryophilic movement. We establish that femtosecond laser ablation can exhibit submicrometer precision, severing individual sensory dendrites without causing collateral damage. We show that severing the dendrites of sensory neurons in young adult worms permanently abolishes their sensory contribution without functional regeneration. We show that the AFD neuron regulates a mechanism for generating cryophilic bias, but we find no evidence that AFD laser surgery reduces a putative ability to generate thermophilic bias. In addition, although disruption of the AIY interneuron causes worms to exhibit cryophilic bias at all temperatures, we find no evidence that laser killing the AIZ interneuron causes thermophilic bias at any temperature.

**Conclusion:**

We conclude that laser surgical analysis of the neural circuit for thermotaxis does not support a model in which AFD opposes cryophilic bias by generating thermophilic bias. Our data supports a model in which the AFD neuron gates a mechanism for generating cryophilic bias.

## Background

A major challenge in understanding the neural basis of complex behavior is determining the functional organization of underlying neural circuits. An advantage of studying simple animals like the nematode *C. elegans *is that quantitative analysis of their movements in response to defined sensory inputs can reveal functions that must be implemented in a nervous system with only 302 neurons. *C. elegans *thermotaxis is particularly interesting as it reveals an interplay of multiple neural functions [1]. Worms adjust a stored set-point of thermotactic memory to their cultivation temperature (*T*_*cult*_). When navigating thermal gradients at temperatures above the thermotactic set-point (*T *> *T*_*cult*_), *C. elegans *crawls down thermal gradients, displaying what is called cryophilic movement. When navigating thermal gradients near the thermotactic set-point (*T ~ T*_*cult*_), *C. elegans *crawls along isotherms. When navigating below the thermotactic set-point (*T *<*T*_*cult*_), we have found that *C. elegans *does not bias its movements either up or down thermal gradients [2]. These features of *C. elegans *thermotaxis show that the underlying neural circuit carries out several functions including: 1) storage of the thermotactic set-point, 2) comparison of current temperature with the thermotactic set-point to determine whether to perform cryophilic movement or isothermal tracking, and 3) measurement of thermal gradients during cryophilic movement or isothermal tracking.

Most behavioral analysis of *C. elegans *thermotaxis has relied on aggregation assays originally developed to screen genetic mutants. Using assays for aggregation at the coldest or warmest points on a thermal gradient, Hedgecock and Russell [1] showed that it was possible to isolate mutants with cryophilic or thermophilic aggregation phenotypes. Mori and Ohshima [3] found that killing certain neurons also leads to cryophilic or thermophilic aggregation. In particular, killing the AFD sensory neuron leads to either cryophilic aggregation or no aggregation (atactic phenotype). Killing the AIY interneuron, which is the main postsynaptic partner of AFD, also leads to cryophilic aggregation. Further, they found that killing the AIZ interneuron, which is postsynaptic to AIY, leads to thermophilic aggregation. A common explanation of cryophilic and thermophilic aggregation is that cryophilic aggregation is caused by damage to a thermophilic pathway, and vice-versa. In this model, thermotactic behavior is explained by different amounts of cryophilic tendency or thermophilic tendency driven by the relative contributions of cryophilic and thermophilic neural pathways.

One problem with the model of separate cryophilic and thermophilic pathways is that wild-type thermotaxis does not appear to involve thermophilic movement in which worms actively migrate up thermal gradients [2]. A satisfactory model of nematode thermotaxis should be framed in terms of neural functions that operate during normal thermotactic behavior. Also, the model of separate cryophilic and thermophilic pathways is framed in terms of cryophilic and thermophilic aggregation, which are behavioral endpoints. The possibility of aggregation mutants means that certain perturbations of the neural circuit – perturbations in the way that the animal transforms sensory input into motor output during thermotaxis – can lead, over time, to the endpoints of cryophilic or thermophilic aggregation. The possibility of such mutants does not necessarily imply the existence of competing neural pathways that target those endpoints.

Here, we address whether AFD function counterbalances cryophilic movement by generating thermophilic movement [4]. The role of the AFD neuron in thermotaxis was originally assigned through systematic laser ablation analysis in combination with assays for cryophilic and thermophilic aggregation [3]. Our reassessment of AFD was motivated by technological developments in both laser surgery and behavioral analysis:

### 1) Laser surgery

Yanik *et al*. [5] demonstrated that tightly-focused femtosecond laser pulses sever nerve fibers in *C. elegans*. The conventional laser ablation technique utilized by Mori and Ohshima [3] employs nanosecond laser pulses to kill the cell bodies of targeted neurons in *C. elegans *larvae, which then develop into adults with behavioral deficits. Nanosecond pulses have sufficient spatial resolution to selectively ablate individual cell bodies, which are several micrometers in size, but their resolution is too coarse to target slender neural structures like sensory dendrites, which have submicrometer diameters and spacing. Here, we extend the application of femtosecond laser ablation, showing that it can be used to sever individual dendrites of sensory neurons, permanently abolishing their sensory contribution in adult worms.

### 2) Behavioral analysis

We developed a microdroplet assay to analyze the thermotactic behavior of individual swimming worms that separates the two thermosensory responses relevant to cryophilic movement [2]. To determine whether to perform cryophilic movement, the worm must decide whether the absolute temperature of its environment is higher or lower than its stored thermotactic set-point. During the performance of cryophilic movement, the worm responds to changes in temperature, generating what we call cryophilic bias by reorienting more (less) frequently in response to warming (cooling). In our microdroplet assay, worms are subjected to sinusoidal temporal variations in temperature at different baseline temperatures. Worms decide to perform cryophilic movement in response to the baseline component of the thermal stimulus (when *T *> *T*_*cult*_). The worm generates cryophilic bias in response to the warming and cooling phases of the thermal stimulus.

In these studies, we combine femtosecond laser surgeries of the thermotactic neural circuit with quantitative behavioral analysis using the microdroplet assay. We quantify the effects of severing the AFD sensory dendrites and of killing the AFD neurons on cryophilic bias. For comparison, we quantify the effect of AFD surgeries in a *ttx-3 *mutant background, which has a genetic disruption of the AIY interneuron. We also quantify the effects of killing the AIZ neurons, which was previously reported to cause thermophilic aggregation [3]. We find no evidence that the AFD neuron can be characterized as part of a thermophilic pathway which counterbalances a separate cryophilic pathway. We favor a model in which the AFD and AIY neurons gate the separate mechanism for generating cryophilic bias based on the thermosensory input of current temperature and the stored thermotactic set-point.

## Results and discussion

### Femtosecond laser ablation

Yanik *et al*. [5] used tightly-focused 50 nJ femtosecond pulses to sever the axons of motor neurons in a study of neuronal regrowth and repair. In order to apply this technology to the sensory dendrites of the AFD neuron, which are tightly bundled with the dendrites of other amphid neurons, we first had to verify that femtosecond laser ablation exhibits submicrometer resolution in *C. elegans*. Shen *et al*. [6] have established that tightly-focused femtosecond laser pulses closer to the ionization threshold (~ 3 nJ in our apparatus) can generate a plasma that is only 300 nm in diameter. The combination of ultrashort duration of the laser pulse, low energy, and high thermal conductivity of an aqueous medium also minimizes the long-range effects of laser irradiation such as mechanical stress, bond-breaking, or heating. For example, calculations show that although the plasma reaches a temperature of 10,000 K, the temperature rise 1 μm from the focal point is at most 10°C and lasts only 2 μs [6]. We sought validation of the submicrometer precision and the permanence of damage by femtosecond laser ablation by visualizing the damage and by testing neuronal function.

**Figure 1:**
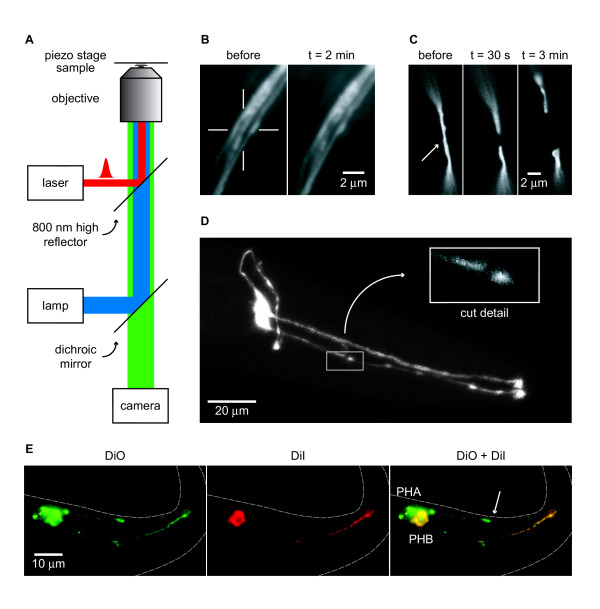
**Femtosecond laser ablation**. **A**. Diagram of laser surgery setup. Amplified femtosecond laser pulses are tightly focused by an objective lens (1.4 NA) to dissect targets positioned with a three-dimensional piezoelectric stage and visualized by fluorescence microscopy. **B**. DiO-stained amphid dendrites before and after severing the middle dendrite using 3-nJ pulses without visibly affecting neighboring dendrites as close as 500 nm away. **C**. An example of a GFP-labeled AFD dendrite retracting after surgery using 6-nJ pulses. **D**. Confocal microscope image showing GFP-labeled AFD neurons the day after surgically severing one dendrite. The position of the cut along the dendrite is representative of all experiments. **E**. Phasmid DiO (pre-surgery) and DiI (post-surgery) staining 24 hours after surgery of the PHA dendrite. The PHA cell body does not absorb DiI because it is physically disconnected from its sensory endings. The cut in the dendrite is indicated with the arrow.

We verified the spatial resolution of femtosecond laser ablation in *C. elegans *by ablating a submicrometer segment from a nerve fiber. A schematic diagram of the apparatus permitting simultaneous fluorescence imaging and femtosecond laser ablation is shown in Fig. 1A. Using the lipophilic fluorescent dye DiO, we stained the subset of amphid neurons that have ciliary projections through openings in the worm's cuticle skin [7]. The fasciculated amphid dendrites are enclosed and separated by sheath cells, so it is straightforward to visualize separate fibers. A single dendrite within the bundle was severed by placing it at the focal point of femtosecond laser pulses. Pulses with energies below the ionization threshold lead to photobleaching at the focal point, which can be distinguished from ablation by the rapid recovery of fluorescence due to diffusion of fluorophore into the intact fiber. Fig. 1B shows the dendritic bundle both before and two minutes after ablation with 3 nJ pulses; after ablation the central dendrite is cut while neighboring dendrites as close as 500 nm are not visibly damaged.

The severed ends of a dendrite often retract from the ablation point by as much as 5 μm. Fig. 1C shows an example of the ends of a severed dendrite labeled with GFP retracting over time. The lack of fluorescent bleed into extracellular compartments or other cells implies that the severed ends reseal after surgery. At lower pulse energies, ablation initiates "pearling", in which the severed dendrite deforms into balls connected by thin filaments. A similar phenomenon has been observed when optical tweezers are focused on artificial tubular membranes [10].

Fluorescence microscopy indicates that the cut persists for at least 24 hours after surgery (Figure 1D). Dye staining experiments provided direct evidence that the cut physically disconnects the cell body from the dendritic tip [7]. We stained the dendrites of the PHA and PHB neurons using the green fluorescent dye DiO, which is absorbed by both neurons through their sensory endings. We severed the dendrite of PHA, and 24 hours later we restained with the red fluorescent dye DiI (Fig. 1E). DiI does not penetrate to the PHA cell body, verifying that laser surgery produces a lasting physical cut. In over 50 operated and re-imaged worms we found that dissected cells remain alive and intact for several days, but in no case did we observe sensory neurons repair themselves by growing a new dendrite, in contrast to a recent report [5].

We also verified the spatial resolution and persistence of femtosecond laser ablation with an assay of osmotic avoidance. The dendrites of the amphid neurons AFD and ASH are bundled together with those of other amphid neurons [8]. The ASH neuron enables the worm to avoid crawling into regions of high osmolarity by sensing ambient osmolarity with the ciliary tips of its dendrites [9]. When wild-type worms are placed in the center of a small high osmolarity ring, the worms retreat from the boundary and are trapped inside the ring. Mutant worms defective in ASH-mediated osmosensation (*osm-3*) do not sense the boundary and rapidly escape from the ring. We examined whether severing the dendrites of the AFD neurons disrupts osmosensory function by collateral damage to the ASH dendrites. Using worms expressing a *gcy-8::gfp *transgene that specifically labels the AFD neuron, we performed surgery to sever the AFD dendrites, and then assayed the worms for osmotic avoidance. We found that after severing both AFD dendrites, worms remain trapped inside the rings to the same extent as normal worms (Table 1). Therefore, severing the AFD dendrites does not disrupt osmosensory function, indicating that the nearby ASH dendrites are not damaged. Finally, we tested the persistence of the cut by assaying osmotic avoidance of worms with severed ASH dendrites at different times after the surgery. ASH dendrites were severed in worms that express a *sra-6::gfp *transgene, which labels the ASH neuron. Severing both dendrites of the ASH neurons disrupts the ability of the worms to sense the high osmolarity ring; these worms do not recover osmosensory function even after 4 days, indicating that the damage is permanent (Table 1).

Our studies, in which severed amphid dendrites do not repair themselves or restore function, contrast with a recent report of axon repair and restoration of motility after severing worm motor neurons using high-energy femtosecond pulses [5]. This variation in outcomes is likely due to differences in the cell biology of worm sensory neurons and worm motor neurons. An analogous difference is found between neurons in the head and body in mammalian neurobiology, that is between the non-regenerating neurons of the central nervous system and the regenerating neurons of the peripheral nervous system [11]. For the purposes of this investigation, our observations indicate that severing worm sensory dendrites using femtosecond laser ablation permanently abolishes their sensory contribution.

### Thermotactic behavior

**Figure 2:**
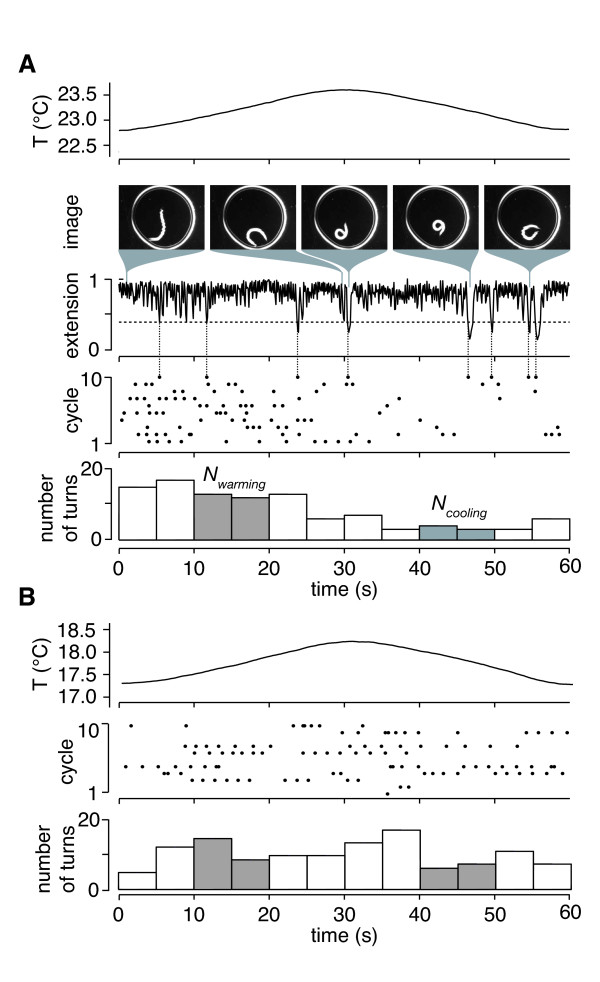
**Microdroplet assay**. **A. **Sample data of cryophilic bias at temperatures above *T*_*cult*_. The top panel shows the sinusoidal thermal cycle recorded at the microdroplet. Sample video frames depict worm conformation and the corresponding body extension; dips of body extension below a threshold are counted as reorientation events. A time series of reorientation events is presented in the fourth panel as a raster of all cycles. The bottom panel is a histogram of reorientation events, showing the distribution of reorientations with respect to the thermal cycle. To avoid sensitivity to possible behavioral phase delays, we use the data from the 10 s in the middle of warming and cooling intervals (dark histogram bars). The tendency to reorient more frequently during the warming phase than the cooling phase of the thermal cycle reveals cryophilic bias. **B. **Sample data of unbiased movement below *T*_*cult*_. Reorientations occur randomly throughout the thermal cycle, showing that this worm is not exhibiting thermophilic or cryophilic bias.

**Figure 3:**
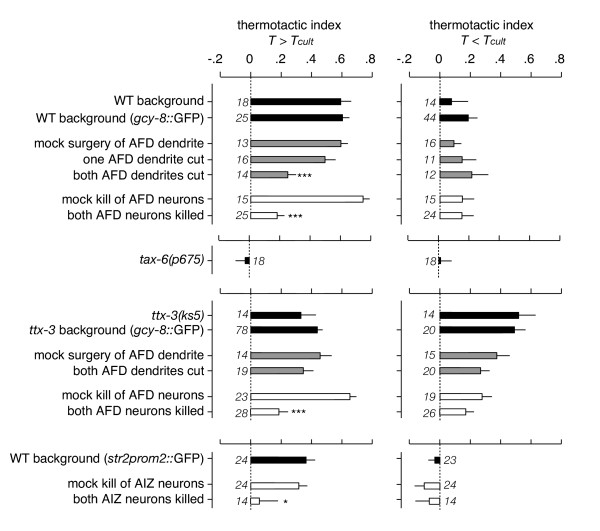
**Effects of laser surgery on cryophilic bias**. The amount of cryophilic bias exhibited by worms assayed above and below *T*_*cult*_. In each panel, black bars correspond to experiments with worms that did not undergo surgery or surgical preparation, gray bars correspond to experiments in which AFD dendrites were severed, and white bars correspond to experiments in which neurons were killed. Mock control measurements represent worms that were prepared for surgery without undergoing laser irradiation. The values of the thermotactic index are means ± one standard error. The cases in which surgery significantly affects cryophilic bias in comparison to its respective mock control are indicated by asterisks (***, *P *< 0.0005; *, *P *< 0.05). All other cases are not distinguishable from their respective mock controls (*P *> 0.2). Significance was measured using a two-tailed Student's *t*-test. The number of worms tested in each measurement of cryophilic bias is noted next to each data bar.

We next sought to investigate the thermosensory contribution of the AFD neuron by analyzing the effects of femtosecond laser surgery on the ability of the worm to bias its movement in response to temperature changes at temperatures above its thermotactic set-point (when worms normally exhibit cryophilic bias, see sample data in Fig. 2) and at temperatures below the set-point (when worms normally do not exhibit cryophilic or thermophilic bias). For all experiments, worms were cultivated at 20°C using standard bacterial food, so that thermotactic behavior at *T *> *T*_*cult *_could be quantified using the microdroplet assay with a thermal cycle between 22.8°C and 23.8°C and at *T *<*T*_*cult *_using a thermal cycle between 17.2°C and 18.2°C. The control data shown in Fig. 3 confirm the contrast in behavior between *T *> *T*_*cult *_and *T *<*T*_*cult *_of wild-type worms. Consistent with our other report using this method, worms do not exhibit thermophilic bias at *T *<*T*_*cult *_[2]. Wild-type worms express the *gcy-8::gfp *transgene exhibit strong cryophilic bias at *T *> *T*_*cult*_, but also exhibit slight cryophilic bias at *T *<*T*_*cult*_, which can be attributed to the presence of the transgene. We tested several transgenic lines before choosing the one with thermotactic behavior closest to wild-type. To separate the effects of femtosecond laser dissection from surgical preparation itself in each experimental run, we compared surgically operated worms with "mock" control groups of worms that underwent surgical preparation without laser irradiation.

We found that severing just one of the two AFD sensory dendrites of the wild-type background strain does not significantly affect thermotactic behavior (Fig. 3). Worms with one severed dendrite exhibit strong cryophilic bias at *T *> *T*_*cult *_and weak cryophilic bias at *T *<*T*_*cult*_, just like worms with intact dendrites. We severed either the right or left dendrite in these surgeries, and found no evidence for asymmetric effects, as might be expected given the electrical gap junction that connects AFDL and AFDR as well as the overall symmetry in their other synaptic connections [8]. However, severing both AFD dendrites leads to uniformly weak cryophilic bias at *T *> *T*_*cult *_and at *T *<*T*_*cult *_in the wild-type background (Fig. 3). For comparison, we studied the effects of killing both AFD neurons. For unknown reasons, young adult worms in the corresponding mock control experiments – which had been prepared for surgery as larvae without laser irradiation – exhibited stronger cryophilic bias at *T *> *T*_*cult *_than wild-type worms. Killing both AFD neurons significantly weakens, but does not eliminate, cryophilic bias at *T *> *T*_*cult *_(Fig. 3). We conclude that either severing both AFD dendrites or killing both AFD neurons leads to thermotactic behavior at *T >T*_*cult *_that resembles behavior at *T *<*T*_*cult*_, i.e., cryophilic bias appears to become unregulated by absolute temperature.

The *tax-6 *gene encodes a calcineurin subunit expressed in the AFD neuron. Kuhara *et al*. found that worms carrying the mutation *tax-6(p675) *exhibit thermophilic aggregation [12]. Kuhara *et al*. argued that the *tax-6(675) *mutation works by hyperactivating the AFD neuron, strengthening the thermophilic pathway, which leads to the thermophilic aggregation phenotype. We analyzed the *tax-6(p675) *mutant in the microdroplet assay, but found no evidence for either cryophilic or thermophilic bias at *T *> *T*_*cult *_or at *T *<*T*_*cult *_(Fig. 3).

The AIY interneuron is the main postsynaptic partner of AFD [8]. Disrupting the AIY interneuron by laser ablation or mutation in the *ttx-3 *LIM homeobox gene leads to cryophilic aggregation [3,13]. In microdroplet experiments with *ttx-3(ks5) *mutants and *ttx-3(ks5) *mutants expressing the *gcy-8::gfp *transgene, we confirmed strong cryophilic biases both at *T *<*T*_*cult *_and at *T *> *T*_*cult *_(Fig. 3). We found that severing both AFD dendrites or killing both AFD neurons tends to weaken but not eliminate the cryophilic bias (Fig. 3). The reduction in cryophilic bias is most evident at *T *> *T*_*cult *_with both AFD neurons killed. These observations suggest that AFD continues to affect the mechanism for generating cryophilic bias even with genetic disruption of AIY by the mutation *ttx-3(ks5)*. Either the disruption of AIY by the mutation *ttx-3 *is incomplete, or AFD transmits behaviorally relevant information through another channel of synaptic output.

Mori and Ohshima [3] found evidence that killing the AIZ interneuron leads to thermophilic aggregation. The interpretation of this effect in terms of the model of separate thermophilic and cryophilic pathways is that killing AIZ damages the cryophilic pathway, leaving the intact thermophilic pathway comprised of AFD and AIY to drive thermophilic aggregation. To kill the AIZ neuron in larval worms by laser ablation, we used worms that express *str2prom2::gfp*, which labels the AIZ neuron. Before surgery these worms exhibited strong cryophilic bias at *T *> *T*_*cult*_, and no significant thermotactic bias at *T *<*T*_*cult*_. However, we found that although killing the AIZ interneuron abolishes cryophilic bias at *T *> *T*_*cult*_, it does not cause thermophilic bias either at *T *> *T*_*cult *_or at *T *<*T*_*cult*_. These data are consistent with a role for AIZ in the mechanism for generating cryophilic bias, but do not support the existence of an alternate thermophilic pathway.

## Conclusion

The surgical effect of snipping the dendrites of the AFD neurons is essentially the same as the surgical effect of killing the AFD neurons. This result suggests that thermosensory measurement by AFD is localized to the distal end of the dendrite. It is not obvious that thermosensory measurement needs to be localized at the sensory endings. Unlike chemosensory measurements, which must be localized to pores in the worm's cuticle skin, thermal conduction allows thermosensory measurements to be carried out, in principle, anywhere inside the worm's body. Genetic evidence also suggests that AFD thermosensation is localized to the dendritic tips: mutations which disrupt the morphology of the ciliated tips of AFD also disrupt thermotactic behavior [14-16]. However, it is unclear whether genetic disruption of the ciliated tips is necessarily to blame for the thermosensory phenotype, or whether such mutations have pleiotropic effects. Severing the AFD dendrite midway between the ciliary tips and the AFD cell body, as depicted in Fig. 1D, isolates any thermosensory measurement at ciliated tips of the AFD dendrite while leaving the AFD cell body, axon, and synaptic architecture intact. This observation also argues against the possibility that the role of AFD in thermotaxis is only to transmit thermosensory signals received from its presynaptic partners; severing the AFD dendrite should only disrupt information transmission from the AFD sensory endings to the cell body, not alter synaptic transmission into or out of AFD.

In our experiments, AFD surgeries consistently reduce cryophilic bias at *T *> *T*_*cult *_without increasing cryophilic bias at *T *<*T*_*cult*_. If AFD were part of a thermophilic pathway, we would have expected AFD surgeries to increase cryophilic bias, especially at *T *<*T*_*cult*_. On the other hand, our observation that AFD surgery weakens without abolishing the mechanism for generating cryophilic bias might explain the variable outcomes of atactic and cryophilic phenotypes after AFD laser killing in the experiments of Mori and Ohshima [3]. Those experiments also reported that killing the AIZ interneuron leads to thermophilic aggregation, and argued that AFD contributes to driving this thermophilic aggregation. Our investigation of AIZ laser killing showed that although killing AIZ abolishes cryophilic bias, killing AIZ does not produce thermophilic bias. In short, this study does not support a model in which *C. elegans *thermotaxis is explained as the output of cryophilic and thermophilic pathways, nor a role for AFD and AIY within a putative thermophilic pathway. This study supports the existence of a mechanism for generating cryophilic bias, and it is possible that AIZ is part of this mechanism.

**Figure 4:**
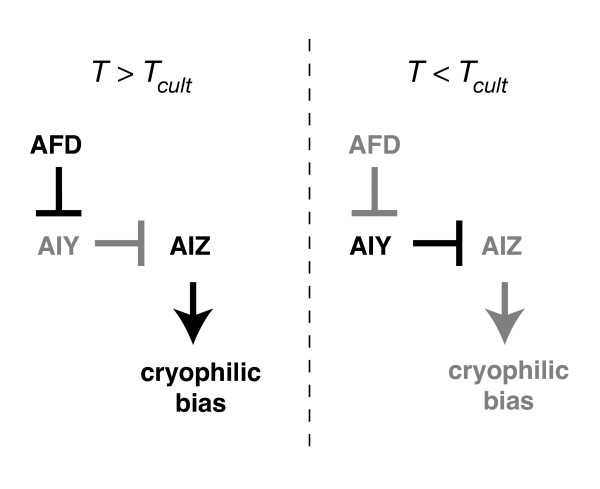
**A gating model for AFD and AIY in generating cryophilic bias**. In this schematic, the synaptic connections between AFD and AIY and between AIY and AIZ are inhibitory, and AIZ contributes directly to generating cryophilic bias. We suggest that patterns in the activity of the neural circuit differ when *T *> *T*_*cult *_and when *T *<*T*_*cult*_. High and low levels of activity are differentiated by black and gray, respectively.

Our study agrees with Mori and Ohshima [3] in that the AFD, AIY, and AIZ neurons contribute to cryophilic movement, but disagrees in the manner of their contribution. We find no evidence that AFD and AIY oppose the mechanism for generating cryophilic bias by generating thermophilic bias. We favor a regulatory model in which AFD and AIY gate the mechanism for generating cryophilic bias. One possibility is that AFD is required to stimulate a mechanism for generating cryophilic bias at *T *> *T*_*cult *_and AIY is required to suppress this mechanism at *T *<*T*_*cult*_. It is also possible that at *T *> *T*_*cult*_, AFD synaptic output to AIY prevents AIY from suppressing the mechanism for generating cryophilic bias via AIZ (a possibility schematized in Fig. 4). Because surgery to AFD weakens cryophilic bias at *T *> *T*_*cult *_even in a *ttx-3 *background, it appears that AFD might also able to stimulate the mechanism for generating cryophilic bias through another channel of synaptic output, either a synapse that has not yet been identified or a gap junction to AIB, the only other output of AFD identified by electron microscopy [8]. In order to evaluate these alternatives, it will be necessary to further map the thermotactic circuit by laser ablation and isolate new thermotactic mutants.

Direct measurements of AFD neuronal activity also illuminate the functional role of AFD in thermotaxis. Kimura *et al*. [17] showed that intracellular calcium dynamics of the AFD neuron are evoked by positive temperature ramps only at absolute temperatures above *T*_*cult*_. The pattern of AFD calcium activity reflects the condition that *T *> *T*_*cult*_, a condition that applies to the normal display of cryophilic bias. A physiological study of spontaneous synaptic release by the AFD neuron showed strong synaptic activity when *T *> *T*_*cult *_or when *T *<*T*_*cult*_, but less at *T ~ T*_*cult *_[18]. The study of synaptic release is more difficult to reconcile with a model in which AFD either stimulates the mechanism for generating cryophilic movement when *T *> *T*_*cult *_or suppresses this mechanism when *T *<*T*_*cult*_, but not both. One possibility is that a more complicated model is required. Another possibility is that the synaptic release observed when *T *<*T*_*cult *_might indicate the artifact of gene-expression in AFD, also encountered in this study, which leads to slight cryophilic bias at *T *<*T*_*cult*_.

Recently, several studies have investigated the roles of neurons in navigational behavior, by systematically disrupting neurons and quantifying the effects on forward movement and reorientation maneuvers [19-21]. In particular, Tsalik and Hobert showed that disruptions of AFD and AIY lower and raise the rate of spontaneous reorientation maneuvers, respectively [18]. One possibility is that AIY acts to inhibit reorientation maneuvers, and that AFD acts to inhibit AIY. Tsalik and Hobert's model of AFD and AIY with respect to reorientation maneuvers bears comparison to our gating model of AFD and AIY with respect to generating cryophilic bias that is schematized in Fig. 4. It is likely that neural circuits operate in similar ways even as they contribute to different aspects of behavior.

It is important to stress the difference between conventional assays of *C. elegans *thermotactic behavior that quantify thermophilic and cryophilic aggregation and our microdroplet assay that quantifies the worm's ability to alter movement patterns in response to positive and negative temperature gradients [1,3]. We interpret the ability of the worm to bias its movements towards lower temperatures, as measured in the microdroplet assay, underlies the worm's ability to crawl down thermal gradients and exhibit cryophilic aggregation in conventional assays. However, it is a current controversy whether *C. elegans *has a capacity to actively crawl up thermal gradients and exhibit thermophilic aggregation [2,22].

The study of behavior in *C. elegans *often begins when genes or neurons are associated with behavioral endpoints like cryophilic aggregation. But in order to understand the functional organization of genes and neurons, the mechanisms that comprise complex behavior must be identified. This effort requires behavioral assays designed to disentangle and analyze each mechanism of complex behavior, and physiological assays to manipulate and monitor neuronal structure and function. In this study, we used femtosecond laser ablation in combination with a quantitative assay of cryophilic bias to clarify the role of the AFD sensory neuron in thermotaxis. Our observations suggest a model in which the AFD neuron regulates cryophilic performance, not by generating thermophilic bias, but by gating a separate mechanism for generating cryophilic bias.

## Methods

### Strains

*C. elegans *strains were cultivated following standard procedures [23]. We used the transgenic strain PY1157, a wild-type N2 background strain with the integrated transgene *gcy-8::gfp *and PY1669, a *ttx-3 (ks5) *mutant background strain with the integrated transgene *gcy-8::gfp*. PY1157, PY1669, and *tax-6(p675) *were gifts of Piali Sengupta, Brandeis University. We also used the transgenic strain OH898, with the transgene *str2prom2::gfp*, which was obtained the *C. elegans *Genetics Center. Wild-type N2 and *osm-3(p802) *were also obtained from the *C. elegans *Genetics Center.

### Femtosecond laser ablation

An amplified Titanium:sapphire laser system emits a 1-kHz train of near infrared pulses (λ = 800 nm), with pulse durations of about 100 fs. By serial insertion of neutral density filters and Kepler telescopic lenses, we adjusted the pulse energy to about 3 nJ at the sample and overfilled the back-aperture of a Zeiss Plan Apochromat 63x, 1.4-NA oil-immersion objective. The same objective was used to image the sample by epifluorescence microscopy using a Coolsnap CCD camera (Roper Scientific Photometrics, Tucson, AZ). The image plane of the CCD camera was adjusted to the laser focus by visualizing the plasma emission created by higher energy laser pulses in coverslip glass.

For femtosecond laser ablation, we followed pre – and postoperative procedures established for standard laser microsurgery [24]. We severed AFD dendrites in transgenic L4 or young adult worms that had been cultivated overnight at 20°C. We killed AFD neurons in L1 worms. We killed AIZ neurons in L2 worms, the larval stage at which the fluorescent label of AIZ became unambiguous. Worms were rinsed in NGM buffer, anaesthetized with 0.2–0.8 mM sodium azide, and mounted on a thin pad of 2% agarose between a glass coverslip and slide. Using fluorescence microscopy and a three-axis piezoelectric nanopositioning stage (Thorlabs MDT630), the target was placed in the focal point of laser pulses and then ablated. Worms were recovered from the sodium azide treatment within 30–60 minutes by rinsing them in NGM buffer for 20 minutes and then placing them on fresh plates with bacterial food. After recovery from sodium azide, worms were immediately returned to the 20°C incubator for at least 12 h before undergoing behavioral assays as young adults.

### Microdroplet assay

Thermotactic behavior was quantified by placing individual worms in a 1-μL droplet sandwiched between a sapphire window and a glass coverslip, subjecting them to 8–10 sinusoidal thermal cycles with a 60-s period using feedback-controlled Peltier-effect thermoelectric elements, and analyzing worm movement using video microscopy [2]. The temperature of the microdroplet was continuously monitored by a 200 – μm diameter thermocouple (Physitemp, Clifton, NJ). Images of the swimming worms were captured by a CCD camera at 10 Hz (Panasonic WV-BP554), and were analyzed in LabVIEW (see Fig. 2).

Swimming motility is characterized by periods of forward swimming punctuated by reorientation events. A worm exhibiting cryophilic bias reorients more frequently during the warming phase than the cooling phase of thermal cycles. By counting the number of reorientation events during the warming and cooling phases of the thermal cycles, *N*_warming _and *N*_cooling_, we can quantify an individual worm's thermotactic behavior by the thermotactic index:

thermotactic index=Nwarming−NcoolingNwarming+Ncooling
 MathType@MTEF@5@5@+=feaafiart1ev1aaatCvAUfKttLearuWrP9MDH5MBPbIqV92AaeXatLxBI9gBaebbnrfifHhDYfgasaacH8akY=wiFfYdH8Gipec8Eeeu0xXdbba9frFj0=OqFfea0dXdd9vqai=hGuQ8kuc9pgc9s8qqaq=dirpe0xb9q8qiLsFr0=vr0=vr0dc8meaabaqaciaacaGaaeqabaqabeGadaaakeaacqqG0baDcqqGObaAcqqGLbqzcqqGYbGCcqqGTbqBcqqGVbWBcqqG0baDcqqGHbqycqqGJbWycqqG0baDcqqGPbqAcqqGJbWycqqGGaaicqqGPbqAcqqGUbGBcqqGKbazcqqGLbqzcqqG4baEcqGH9aqpdaWcaaqaaiabd6eaonaaBaaaleaacqqG3bWDcqqGHbqycqqGYbGCcqqGTbqBcqqGPbqAcqqGUbGBcqqGNbWzaeqaaOGaeyOeI0IaemOta40aaSbaaSqaaiabbogaJjabb+gaVjabb+gaVjabbYgaSjabbMgaPjabb6gaUjabbEgaNbqabaaakeaacqWGobGtdaWgaaWcbaGaee4DaCNaeeyyaeMaeeOCaiNaeeyBa0MaeeyAaKMaeeOBa4Maee4zaCgabeaakiabgUcaRiabd6eaonaaBaaaleaacqqGJbWycqqGVbWBcqqGVbWBcqqGSbaBcqqGPbqAcqqGUbGBcqqGNbWzaeqaaaaaaaa@7333@

This thermotactic index varies from -1 for a thermophilic worm that only reorients during the cooling phase of a thermal cycle to +1 for a cryophilic worm that only reorients during the warming phase.

We used a machine-vision algorithm for flagging turns in video records of swimming worms. Forward swimming and turns can be distinguished using an "extension factor" assigned to the worm's conformation in each video frame. During forward swimming, the worm body has an extended conformation as bending waves travel rhythmically from nose to tail. Each turn, corresponding to a sudden sharp bend, can be automatically detected as dips in body extension below a threshold value. For automated and objective analysis, an algorithm sets the extension threshold so that each worm has the same average reorientation rate – because the thermotactic index is sensitive to the distribution of reorientation events in the cycle and not to the overall number of events in each cycle, this step does not affect the value of the thermotactic index when worms exhibit different average rates of reorientation, but does automate the setting of the extension threshold in each experiment.

### Ring assay of the osmosensory behavior

Worms were assayed for ASH osmosensory function after surgery or mock surgery following standard procedures [9]. Approximately 20 μL of NGM or 8 M glycerol was distributed in a 15 mm diameter ring on an agar plate and allowed to dry. Young adult worms were rinsed with NGM buffer, allowed to crawl for several minutes on an unseeded agar plate, and then picked to the center of normal (NGM) or high (glycerol) osmolarity rings. We scored the number to escape from the rings after 10 min.

## Authors' contributions

SHC performed femtosecond laser ablation surgeries and osmolarity avoidance assays. DAC and CVG performed laser surgeries, microdroplet behavioral assays, and analyzed data. SHC, DAC, CVG, EM, and ADTS designed the study and drafted the manuscript. All authors read and approved final manuscript.

## Figures and Tables

**Table 1:** Effects of severing AFD and ASH dendrites on ASH-specific osmosensory function

Strain	Surgery	**Hours after surgery**	**% Escape NGM Rings (*n*)**	**% Escape Glycerol Rings (*n*)**
*osm-3(p802)*	--	--	90 ± 5 (*30*)	83 ± 7 (*30*)
WT (*gcy-8::gfp*)	--	--	93 ± 5 (*30*)	3 ± 3 (*30*)
	mock	24	83 ± 7 (*29*)	0 ± 0**(*31*)
	both AFD dendrites cut	24	84 ± 7 (*25*)	
WT (*sra-6::gfp*)	mock	24	92 ± 6 (*24*)	0 ± 0**(*24*)
	both ASH dendrites cut	24	100 ± 0 (*26*)	77 ± 8(*26*)
	mock	48	100 ± 0 (*8*)	0 ± 0**(*8*)
	both ASH dendrites cut	48	88 ± 8 (*17*)	83 ± 9(*18*)
	mock	72	100 ± 0 (*24*)	0 ± 0**(*24*)
	both ASH dendrites cut	72	100 ± 0 (*15*)	93 ± 6(*15*)
	mock	96	86 ± 8 (*21*)	0 ± 0** (*21*)
	both ASH dendrites cut	96	77 ± 9 (*22*)	73 ± 9 (*22*)

Comparison of ASH-mediated osmosensory function in (*i*) an *osm-3 *mutant strain that lacks ASH function, (*ii*) a wild-type background strain with or without both AFD dendrites cut, 24 hours after surgery, and (*iii*) a wild-type background strain with the ASH neuron expressing GFP with and without both ASH dendrites cut, 24, 48, 72, and 96 hours after surgery. We assayed the ability of worms to sense the boundaries of rings of glycerol solution or NGM buffer on an agar surface one day after undergoing surgery or mock surgery. We report the percentage ± standard error of escape from the rings after 10 min. There is no significant difference between the escape percentages between different worm strains in the column corresponding to NGM rings. For each row, asterisks denote significant difference between escape from glycerol rings and from NGM rings (** *P *< 10^-5^).

## References

[B1] Hedgecock EM, Russell RL (1975). Normal and mutant thermotaxis in the nematode *Caenorhabditis elegans*. Proc Natl Acad Sci USA.

[B2] Ryu WS, Samuel AD (2002). Thermotaxis in *Caenorhabditis elegans *analyzed by measuring responses to defined thermal stimuli. J Neurosci.

[B3] Mori I, Ohshima Y (1995). Neural regulation of thermotaxis in *Caenorhabditis elegans*. Nature.

[B4] de Bono M, Maricq AV (2004). Neuronal substrates of complex behaviors in C. elegans. Ann Rev Neurosci.

[B5] Yanik MF, Cinar H, Cinar HN, Chisholm AD, Jin Y, Ben-Yakar A (2004). Neurosurgery: functional regeneration after laser axotomy. Nature.

[B6] Shen N, Datta D, Schaffer CB, LeDuc P, Ingber DE, Mazur E (2005). Ablation of cytoskeletal filaments and mitochondria in cells using a femtosecond laser nanoscissor. Mech Chem Biosys.

[B7] Hedgecock EM, Culotti JG, Thomson JN, Perkins LA (1985). Axonal guidance mutants of *Caenorhabditis elegans *identified by filling sensory neurons with fluorescein dyes. Dev Biol.

[B8] White JG, Southgate E, Thomson JN, Brenner S (1986). The structure of the nervous system of the nematode *C. elegans*. Philos Trans R Soc Lond B Biol Sci.

[B9] Hart AC, Kass J, Shapiro JE, Kaplan JM (1999). Distinct signaling pathways mediate touch and osmosensory responses in a polymodal sensory neuron. J Neurosci.

[B10] Bar-Ziv R, Moses E (1994). Instability and "pearling" states produced in tubular membranes by competition of curvature and tension. Phys Rev Lett.

[B11] Case LC, Tessier-Lavigne M (2005). Regeneration of the adult central nervous system. Curr Biol.

[B12] Kuhara A, Inada H, Katsura I, Mori I (2002). Negative regulation and gain control of sensory neurons by the *C. elegans *calcineurin TAX-6. Neuron.

[B13] Hobert O, Mori I, Yamashita Y, Honda H, Ohshima Y, Lui Y, Ruvkun G (1997). Regulation of interneuron function in the *C. elegans *thermoregulatory pathway by the *ttx-3 *LIM homeobox gene. Neuron.

[B14] Satterlee JS, Sasakura H, Kuhara A, Berkeley M, Mori I, Sengupta P (2001). Specification of thermosensory neuron fate in *C. elegans *requires *ttx-1*, a homolog of otd/Otx. Neuron.

[B15] Cassata G, Kagoshima H, Andachi Y, Kohara Y, Dürrenberger MB, Hall DH, Bürglin TR (2000). The LIM Homeobox Gene ceh-14 Confers Thermosensory Function to the AFD Neurons in *Caenorhabditis elegans*. Neuron.

[B16] Perkins LA, Hedgecock EM, Thomson JN, Cullotti JG (1986). Mutant sensory cilia in the nematode *Caenorhabditis elegans*. Dev Biol.

[B17] Kimura KD, Miyawaki A, Matsumoto K, Mori I (2005). The *C. elegans *thermosensory neuron AFD responds to warming. Curr Biol.

[B18] Samuel AD, Silva RA, Murthy VN (2003). Synaptic activity of the AFD neuron in *Caenorhabditis elegans *correlates with thermotactic memory. J Neurosci.

[B19] Tsalik EL, Hobert O (2003). Functional mapping of neurons that control locomotory behavior in *Caenorhabditis elegans*. J Neurobiol.

[B20] Gray JM, Hill JJ, Bargmann CI (2005). A circuit for navigation in *Caenorhabditis elegans*. PNAS.

[B21] Wakabayashi T, Kitagawa I, Shingai R (2004). Neurons regulating the duration of forward movement in *Caenorhabditis elegans*. Neurosci Res.

[B22] Ito H, Inada H, Mori I (2006). Quantitative analysis of thermotaxis in the nematode Caenorhabditis elegans. J Neurosci Methods.

[B23] Brenner S (1974). The genetics of *Caenorhabditis elegans*. Genetics.

[B24] Bargmann CI, Avery L (1995). Laser killing of cells in *Caenorhabditis elegans*. Methods Cell Biol.

